# Snake Venom Cytotoxins, Phospholipase A_2_s, and Zn^2+^-dependent Metalloproteinases: Mechanisms of Action and Pharmacological Relevance

**DOI:** 10.4172/2161-0495.1000181

**Published:** 2014-01-25

**Authors:** Sardar E Gasanov, Ruben K Dagda, Eppie D Rael

**Affiliations:** 1 Applied Mathematics and Informatics Department, Moscow State University Branch, 22 A. Timur Avenue, Tashkent 100060, Uzbekistan; 2 Science Department, Tashkent Ulugbek International School, 5-A J. Shoshiy Street, Tashkent 100100, Uzbekistan; 3 Pharmacology Department, University of Nevada School of Medicine, 1664 North Virginia St., Reno, NV 89557, USA; 4 Department of Biological Sciences, University of Texas at El Paso, 500 West University Avenue, El Paso, TX 79968, USA

## Abstract

Snake venom toxins are responsible for causing severe pathology and toxicity following envenomation including necrosis, apoptosis, neurotoxicity, myotoxicity, cardiotoxicity, profuse hemorrhage, and disruption of blood homeostasis. Clinically, snake venom toxins therefore represent a significant hazard to snakebite victims which underscores the need to produce more efficient anti-venom. Some snake venom toxins, however, have great potential as drugs for treating human diseases. In this review, we discuss the biochemistry, structure/function, and pathology induced by snake venom toxins on human tissue. We provide a broad overview of cobra venom cytotoxins, catalytically active and inactive phospholipase A_2_s (PLA_2_s), and Zn^2+^-dependent metalloproteinases. We also propose biomedical applications whereby snake venom toxins can be employed for treating human diseases. Cobra venom cytotoxins, for example, may be utilized as anti-cancer agents since they are efficient at destroying certain types of cancer cells including leukemia. Additionally, increasing our understanding of the molecular mechanism(s) by which snake venom PLA_2_s promote hydrolysis of cell membrane phospholipids can give insight into the underlying biomedical implications for treating autoimmune disorders that are caused by dysregulated endogenous PLA_2_ activity. Lastly, we provide an exhaustive overview of snake venom Zn^2+^-dependent metalloproteinases and suggest ways by which these enzymes can be engineered for treating deep vein thrombosis and neurodegenerative disorders.

## Introduction

Snake venom is a complex mixture of organic compounds [1]. Many of these compounds produce a variety of pathophysiological effects including local tissue damage and/or systemic effects in the affected individual [1–4]. The major types of biomolecules found in snake venom are proteins, some of which are enzymes whereas other proteins lack catalytic activity. The most potent toxins of snake venom, which are responsible for causing severe pathophysiological effects after envenomation, are α-neurotoxins – non-enzymatic nAChRs binding proteins [5,6], β-neurotoxins – pre-synaptic phospholipase A2 (PLA2) [7–9] and pre-synaptic phospholipase A_2_ (PLA_2_), β-neurotoxins [7–9], cytotoxins [10–16] non-neurotoxic PLA_2_s [17–20], and Zn^2+^-dependent metalloproteinases [21]. All of these toxins will be briefly reviewed in this section and more extensively in the ensuing sections of this review.

## Epidemiology and Treatment of Snake Envenomation

Worldwide, it is estimated that approximately 2,500,000 human beings suffer from snakebites per year which result in over 125,000 deaths worldwide [22–24]. Most lethal snakebites predominantly occur in Africa and Asia and are caused by approximately 410 venomous species of snakes [22,24]. Clinically, administering anti-venom to the affected patient within a very limited time frame (<2 hours) efficiently reverses many of the detrimental systemic effects caused by snake venom including nephrotoxicity, myotoxicity, and necrosis. Depending on the composition of snake venom, patients may be cotreated with atropine followed by acetylcholinesterase inhibitors in case that neurotoxicity is induced by muscarinic receptor inhibitors. Following anti-venom treatment, the patient is hydrated, and necrotic tissue can be removed by debridement [23]. However, it is widely recognized by many clinicians and toxinologists that administering broad-spectrum antisera may not be the most efficient way for treating snakebite victims due to the highly diverse pharmacological properties of snake venom across snake species [25–27]. Therefore, generating new anti-venom based on purified recombinant toxins, geographic location of snake species that produce the envenomation, and severity of pathology may be the best alternative for treating snake bite victims. On the other hand, limited research and limited federal-supported funding for anti-venom research has hampered the availability and diversity of efficient anti-venom.

Snake venom α-neurotoxins, which are non-enzymatic, three-fingered fold proteins, block nicotinic acetylcholine receptors (nAChR) of muscle and are widely used in nAChR studies [6]. However, the pharmacological relevance of snake venom α-neurotoxins has not yet been clearly defined. The mechanism of action of pre-synaptic PLA_2_ β-neurotoxins, as opposed to α-neurotoxins, is also not well understood, although hydrolysis of phosphatidylcholine (PC), phosphatidylserine (PS), and phosphatidylethanolamine (PE) in neuronal membranes appears to be required for the onset of pre-synaptic neurotoxicity [19]. Hence, a clear understanding of the modulating mechanism(s) of pre-synaptic PLA_2_ β-neurotoxin activities is important for designing drugs for preventing neurodegeneration caused by increased levels of lysophospholipids and fatty acids in the plasma membranes of the synaptic cleft, which likely results from pathological activation of endogenous lipases at the nerve terminals [18].

The mode of action of snake venom cytotoxins has been characterized based on studies of cytotoxins derived from cobra venom. These cytotoxins kill cells by non-selectively disrupting cell membranes [12,14]. Cytotoxins exhibit various physiological effects which are mainly regulated by modifying cell membrane structure and function [2,12,14,28,29]. Various pharmacological applications have been suggested for cytotoxins such as their ability to destroy various types of cancer cells [2,12,14,30].

The non-obligatory protein-binding partners of cytotoxins in snake venom are the catalytically active PLA_2_s. These PLA_2_s promote Ca^+2^-dependent hydrolysis of the 2-acyl ester bond in phosphoglycerides with the consequent release of free fatty acids and lysophospholipids. These PLA_2_s share structural features with endogenous mammalian PLA_2_s that are present in inflammatory exudates in mammals [31,32]. Additionally, snake venom PLA_2_s display numerous pharmacological activities including myotoxic, neurotoxic, anticoagulant, hypotensive, hemolytic, anti-platelet aggregating activity, bactericidal, and pro-inflammatory activities [33–36]. Snake venom PLA_2_s are also able to stimulate neutrophil chemotaxis, degranulate mast cells *in vitro*, and activate macrophages [36].

PLA_2_s are commonly used as enzyme models to study mechanisms that modulate phospholipid hydrolysis [15,37,38]. Given that overactive lipid hydrolysis by endogenous phospholipases stimulates chronic inflammation, there is considerable interest by the medical community for finding ways to decrease PLA_2_ activity in chronic inflammatory conditions like rheumatoid arthritis, asthma, and autoimmune disorders.

Other major constituents of snake venom are the snake venom hemorrhagic metalloproteinases (SVMPs). Most of the Zn^2+^-dependent enzymes, which vary structurally, induces hemorrhage and some have the ability to degrade protein aggregates like fibrin clots. Thus some of these SVMPs may have applications for treating human conditions involving abnormal blood clot formation. For example, alfimeprase a recombinant, truncated version of fibrolase, which was isolated from *Agkistrodon contortrix* venom, has been tested in Phase III clinical trials for the treatment of peripheral arterial occlusive disease and stroke [39].

Given the high variation in their proteolytic activities and their ability to degrade protein aggregates, some SVMPs may be engineered in the near future for degrading insoluble extracellular protein aggregates like β-amyloid and α-synuclein, two forms of extracellular protein aggregates that represent the major instigators for mediating neurodegeneration in advanced Alzheimer’s disease (AD) and Parkinson’s disease (PD) patients.

This review focuses on the latest molecular mechanisms and structure/function studies of snake venom components including membrane-active cytotoxins, catalytically active PLA_2_s, and Zn^2+^-dependent metalloproteinases. We also propose modifying snake venom enzymes for potential biomedical applications.

## Cytotoxins

Snake venom cytotoxins are highly basic amphipatic proteins and they constitute as much as 40–70% of cobra venom (*Naja* and *Haemachatus*). The methods used in their initial detection led to the assignment of descriptive names to these toxins like direct lytic factors, cardiotoxins, cobramines, cytolysins, and membranotoxins [14,40,41]. The mechanisms for cytotoxin-mediated toxicity include modulating the activity of membrane-bound enzymes, depolarizing excitable membranes of heart cells and of neurons, inhibiting platelet aggregation, inducing hemolysis and cytotoxicity, and bringing about cardiac arrest [12,42,43]. It is widely accepted that most pathological activities of cytotoxins are based on their ability to bind to cell membranes leading to alterations in the organization and function of lipid bilayers [2,10,12,14,29,44]. Snake envenomation due to *Naja* species is highly prevalent in Africa and mortality is often associated with the potent necrotic activity of *Naja*-derived cytotoxins. Pathologically, cytotoxins are responsible for severe myotoxicity, hemolysis, and necrosis in the affected human. Clinically, treating snakebite victims in Africa with clinically-approved anti-sera can efficiently reverse pathology by partially immunodepleting cytotoxins found in some *Naja* venoms. Hence, future research endeavors are required for producing promising efficient anti-venom with more potent anti-cytotoxin immunodepleting activities for use in anti-venom clinics [45].

### Structure of cytotoxins

**Figure 1:**
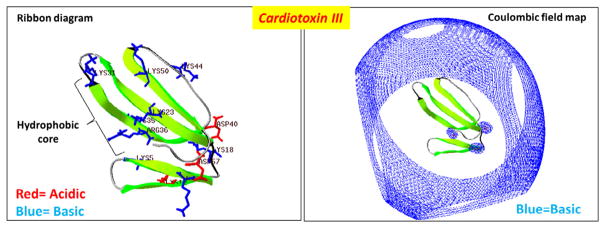
A rendering of the 3D structure of cardiotoxin III from *Naja naja atra* A ribbon diagram of the crystal structure of cardiotoxin III (PDB #2CRT) is illustrated to highlight the hydrophobic core region and show basic residues in blue and acidic residues in red. Right panel: A rendering of the Coloumbic electrostatic field potential map for cardiotoxin III which shows that the toxin possesses a very extensive and wide basic electrostatic field potential landscape (blue). Electrostatic field potential calculations, 3D molecular rendering, and specific annotations of cardiotoxin III was performed by using the Swiss PDB Viewer software.

Cytotoxins contain approximately 60 amino acid residues (~7 kDa) and are structurally characterized by the presence of the three-fingered (TF) fold (Figure 1). The β-strands of the antiparallel β-sheet give rise to the hydrophobic core of the molecule, hence the name of the TF fold. All cobra venom cytotoxins have a similar 3D structure stabilized by four disulfide bonds. Cytotoxins exhibit strong amphiphilic properties on their molecular surface: apolar tips of loops I–III that form a hydrophobic core flanked by a positively charged “ring” composed mostly of conserved Lys and Arg residues that are clustered near the N-terminal region of the protein and by a less polar C-terminal region (Figure 1). In general, most cytotoxins are highly positively charged molecules that have very extensive basic electrostatic field potential maps.

### Molecular mechanisms of toxicity of cytotoxins

Cytotoxins are non-enzymatic toxins and their mode of cytotoxicity involves their ability to form pores on cell membranes. It is believed that the basic electrostatic field potential landscape enables cytotoxins to seek and interact with anionic phospholipids through long distance electrostatic potentials (Figure 1). Several *in vitro* experiments using model membranes [2,14,46–48] have shown that the hydrophobic core of cytotoxins represents the principal membrane-binding motif, and upon binding, cytotoxins produce structural defects in lipid bilayers. This event leads to formation of pores, whose size and life time have been estimated to be in the range of 30 Å and 10^−2^ s respectively [49,50]. Although the precise molecular mechanism of action remains to be elucidated, it is well accepted that basic residues, namely Lys and Arg, are required for the interaction of cytotoxins with negatively charged phospholipids that are located on the outer leaflets of lipid bilayers [51] (Figure 1). While α-neurotoxins (also TF proteins from cobra venom) mainly exert their pathological effects by physically interacting with select protein receptors, no specific protein targets have yet been identified for cytotoxins from cobra venom [29].

Cytotoxins exhibit activity on various cell types, including erythrocytes, lymphocytes, cardiac myocytes, spleen cells, and various tumor cells [14,37,38,52–54]. The pathological consequences, however, depends on the cell membrane proteins and phospholipids found on the outer leaflet of the plasma membrane of these cell types [55]. Cytolysis is considered to be the general mechanism of action [50]. Indeed, cytotoxins possess high affinity for binding to negatively charged lipids [14,46–48,55,56]. Some cytotoxins undergo lipid-induced oligomerization [49]. The interaction of cytotoxins with negatively charged lipids can cause the dimerization of cytotoxins, which eventually leads to oligomerization, a critical step towards the formation of membrane pores. Hence, the oligomerization of cytotoxins is the molecular basis for the formation of membrane pores [42] in a manner similar to that of perforins released by cytotoxic T cells and by natural killer cells. Once thought to exist only as monomers, cytotoxins are now known to form dimers, which can synergize downstream pathology on targeted cells. A neurotoxic dimer containing a cytotoxin was isolated from *Naja kaouthia* venom [57]. This dimer consists of a three-fingered α-neurotoxin bound to cytotoxin via disulfide bonds. The cytolytic activity of the cytotoxin is completely lost upon its association to α-neurotoxin [57].

Some cytotoxins, also known as cardiotoxins, depolarize cardiac myocytes [58,59]. The mechanism of cytotoxicity induced by cardiotoxins in heart cells mainly involves the opening of voltage-dependent Ca^+2^ channels, leading to a block of the inwardly rectifying K^+^ channels and the formation of new abnormal ion conductive pathways [12,59]. It has been suggested that these cytotoxins interact with protein targets in the membrane of cardiac myocytes [42].

### Anti-cancer applications of cytotoxins

The cytotoxin from *N. atra* is cytotoxic to cancer cell lines (MCF-7, P388, K562, and H22) and to normal 16HBE human cells as well [30]. The rank of cytotoxin-induced cell death on these cell lines is as follows: MCF-7>P388 ≈ K562>H22 ≈ 16HBE. Cytotoxins decreased cell viability, promoted apoptosis, and induced lysosomal permeability in a dose- and time-dependent manner. In general, all intoxicated cells showed morphological and biochemical features characteristic of late apoptosis and necrosis. P388 cells treated with cytotoxin lost mitochondrial integrity and function. Cytotoxin can also induce an increase in both lysosomal membrane permeability and cathepsin B-mediated protease activity. Hence, cytotoxins possess significant and selective anticancer activity by inducing programmed cell death through the activation of mitochondrial-mediated cell death pathways and inactivation of the lysosomes [30].

By using confocal spectral imaging, it was shown that cytotoxins from *N. oxiana*, *N. kaouthia,* and *N. haje* can readily penetrate into living cancer cells (i.e., human lung adenocarcinoma A549 and promyelocytic leukemia HL60) and markedly accumulate in lysosomes [11]. Although it was once thought that the main cytotoxic mechanism of cobra cytotoxins involved the disruption of the phospholipid membrane structure [18], few studies have shown [11] that some cytotoxins can be internalized through a non-canonical pathway to produce pathology. The internalization of cytotoxins likely explains the higher sensitivity of some tumor cells to cytotoxin-induced toxicity compared to normal human cells. Cytotoxins do not appear to form stable structures with the cell membrane of A549 cells but are readily internalized. The interaction of cytotoxins from *N. kaouthia* with cell membranes is transient as cytotoxins isolated from this cobra venom were efficiently internalized in HL60 cells while little internalization was seen for cytotoxins isolated from other cobra venom. Moreover, the kinetics on the lysosomal accumulation of cytotoxins correlated well with their cytotoxic effect. Overall, it appears that lysosomes are the major targets of cytotoxins by causing lysosomal leakage whereas plasma membrane injury is a pathological event that followed lysosomal rupture [11].

It is known that tumor cells have a high propensity for internalizing exogenous ligands. This phenomenon partly explains the selective toxicity of cytotoxins for tumor cells and supports a model that involves an initial association of cytotoxins to unknown protein targets on the cell membrane prior to their internalization [60,61]. Following their internalization, cytotoxins can cause significant devastating pathological effects in cancer cells. At the cytosol, both cytotoxins and lysosomal proteases liberated by cytotoxin-induced lysosomal leakage trigger necrosis and plasma cell membrane permeabilization [12]. Cytotoxins can also sequester ATP in the cytosol and disturb ATP-dependent cellular processes resulting in metabolic catastrophe of cancer cells. Cytotoxins can also penetrate the nucleus and bind with chromatin and with relaxed DNA, which also triggers apoptosis [12].

Cytotoxins can also affect the action of various cell membrane channels and receptors including Na^+^/K^+^-ATPase, and integrin receptors [62]. NMR and X-ray studies have shown that cytotoxins can bind with low molecular weight ligands such as heparin-derived oligosaccharides [63,64], nucleotide triphosphates [65], and sulfatide [66]. However, it is important to point out that the distribution of glycosphingolipids in membranes of various mammalian cell types differs greatly which can affect the ability of cytotoxins to bind cell membranes [67]. Considering the extensive pharmacological and biochemical profiles of cytotoxins, it is reasonable to propose that cytotoxins specifically interact with other anionic compounds including anionic lipids (ie, phosphatidylserine, cardiolipin and phosphatidic acid). Phosphatidylserine (PS) is the most abundant anionic phospholipid in mammalian cells and is the major phospholipid constituent of cell membranes that facilitates interfacial contacts with other cell membrane proteins [68,69]. PS exposed on the cell surface is a hallmark of apoptosis and triggers the removal of the cell by phagocytes [70]. Despite the fact that PS is normally located in the inner leaflet of the lipid bilayer, various cellular events including cell activation, membrane fusion, cell division, and aging lead to PS exposure on the cell surface [70–72]. The externalized PS may induce a variety of pathological consequences including malignant cell transformation, cell injury, and infection [71–73]. The inhibition of protein kinase C (PKC) by cytotoxins is a consequence of the association of cytotoxins to a site on PS that is required for the association and activation of PKC [74]. A recent study that used computer simulations to study the interaction between *N. kaouthia* cytotoxin and PS suggest that cytotoxins contain several PS-binding sites [10]. These binding sites have a strong ability to accommodate low molecular weight compounds (like the head group of PS) in a variety of different conformations [10]. Hence, the existence of multiple binding sites for different anionic phospholipids in cytotoxins may likely contribute to variable pharmacological effects and pathology in various types of tissues [10].

The ability of monomeric cytotoxin to discriminate between normal and cancer cells is both intriguing and controversial [14]. However, a plausible biotechnological approach for synthesizing anti-cancer versions of cytotoxins is to conjugate them to monoclonal antibodies that bind to specific epitopes found exclusively on malignant cells. For example, the cytotoxin-like membrane active toxin Pyrulariathionin [75] conjugated to an anti-CD5 monoclonal antibody was very effective in the selective destruction of CD5 positive human lymphocytes *in vitro* [76]. Cytotoxins from *Naja* venoms have also been studied as potential candidates for generating potent novel immunotoxins (unpublished).

### Mechanism of cytotoxin-mediated cellular pathology

In further support of the concept that cytotoxins interact with phospholipids, our lab group has been unable to show that *N. oxiana* cytotoxins Vc5 and Vc1 interact with cell membrane proteins [77,78]. On the other hand, we found that cytotoxin Vc5 indirectly modulates the activity of H^+^-ATP synthase by inducing structural transitions of mitochondrial lipid bilayers [78]. These lipid phase transitions likely occur due to the ability of cytotoxins to bind with phospholipids that are tightly bound and clustered near the F_0_ subunit of ATP synthase. By studying the interaction of cytotoxins with phosphatidylcholine membranes containing small amounts of anionic phospholipids, we unveiled novel mechanisms by which cytotoxins damage cell membranes including their ability to dehydrate the lipid bilayers [47], induce the rapid aggregation of liposomes, increase cell membrane permeability [14], induce the formation of abnormal non-bilayer structures [46,47,79,80], cause an intermembrane exchange with lipids [46–48], or cause cell membranes to fuse [16,81,82]. We have also shown that the anti-cancer activity of Vc5 in Jurkat cells involves its ability to bind with acidic phospholipids [14,77]. Vc5 binds to acidic phospholipids with high affinity (K_d_< 10^−7^ M) with a stoichiometry of approximately eight phospholipid molecules for every toxin molecule [77].

It is important to be aware that different results can be obtained when trace amounts of PLA_2_, which may be present in commercial preparations of cytotoxins [83], are introduced into experimental samples and can confound the interpretation of the results given that cytotoxin bound to PLA_2_ is equally toxic to both normal lymphocytes and Jurkat cells [14]. This observation suggests that cytotoxins can form stable complexes with PLA_2_s. We have previously suggested a mechanism for the cytotoxic synergy produced when PLA_2_s are bound to cytotoxins. In brief, our model suggests that cytotoxin forms a complex with PLA_2_ at neutral pH [14,84]. The cytotoxin-PLA_2_ complex binds transiently to cell membranes, presumably due to the ability of cytotoxin to bind to unknown membrane proteins or acidic phospholipids, which allows PLA_2_ to hydrolyze phospholipids. As a consequence, the hydrophilic regions of cytotoxins become surrounded by released fatty acid tails and these molecular interactions allow cytotoxin to be liberated from the enzyme-toxin complex [84]. Both the phase segregation of fatty acids and an accumulation of lysophospholipids induced by cytotoxins destabilize lipid bilayers and promote the flip-flopping of phospholipids. These events change the initial asymmetric distribution of membrane lipids resulting in the exposure of anionic lipids on the outer membrane leaflet of the cell membrane. These molecular events not only facilitates the interaction of monomeric cytotoxins with anionic lipids on the extracellular side, but also triggers lipase activity on the cytoplasmic side of the cell membrane which exacerbates their destruction [84]. Cytotoxin-induced stimulation of endogenous lipase activity has been well documented [85]. However, it remains to be resolved whether the phospholipase activity that is stimulated as a result of the interaction of cytotoxins with cell membranes is due to endogenous PLA_2_ or phospholipase C (PLC) [85].

**Figure 2:**
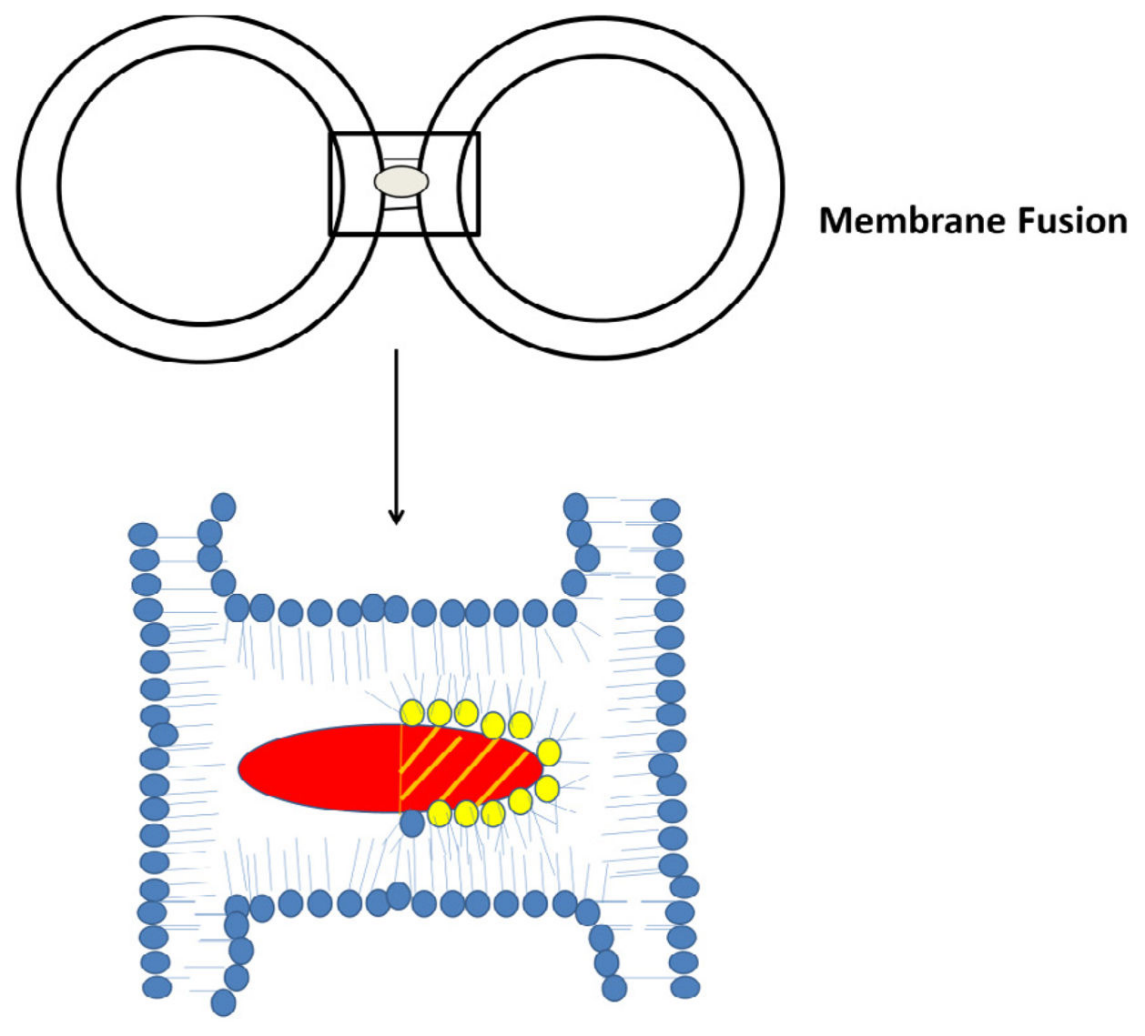
**A** schematic diagram of a model by which cardiotoxin Vc5 enables the fusion of liposomes. Vc5 can simultaneously interact with two liposomes containing a mixture of phosphatidylcholine and cardiolipin by interacting with the outer leaflet of each liposome. Phospholipids shown in yellow are anionic phospholipids (PS and CL). Please note that the hydrophilic region of cardiotoxin (basic N-terminal region; shaded region of cardiotoxin) can sequester either PS or CL molecules away (yellow phospholipid heads) from one of the leaflets of the lipid bilayer while the hydrophobic region of cytotoxin interacts with the alkyl tails of the phospholipids.

**Figure 3:**
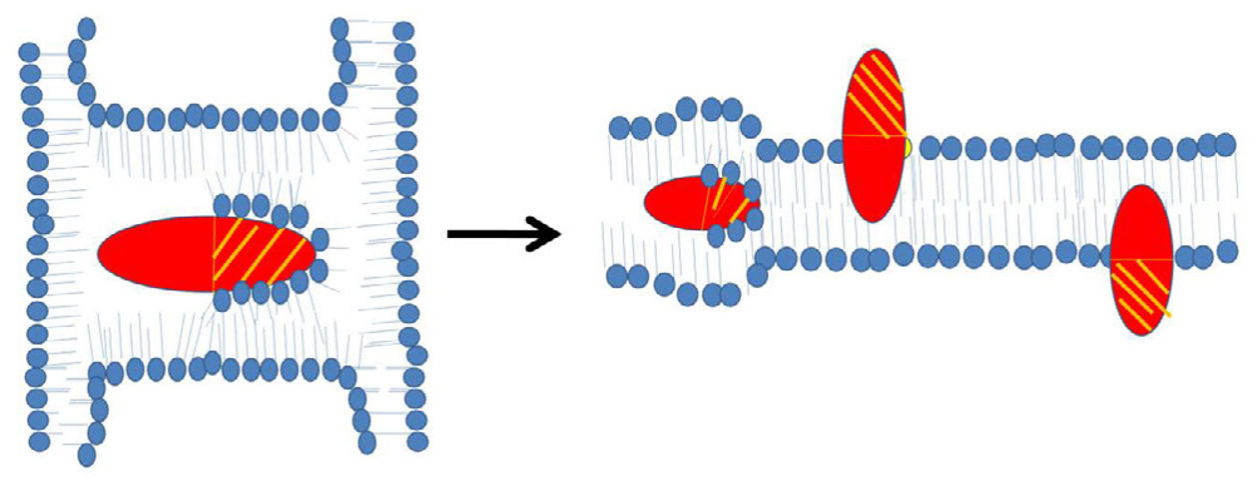
Schematic diagram of a model by which cardiotoxins can form inverted micelles and translocate inside lipid bilayers. Cardiotoxin can imbed inside an inverted micelle (left panel) when trapped between two liposomes or two lipid bilayers (Figure 2). Cardiotoxins can also imbed inside cell membranes to form a transient inverted micelle from which the cytotoxin translocates to either the outer or the inner leaflet of the lipid bilayer with the hydrophilic portion (shaded) exposed to the bulk solvent while the hydrophobic region (non-shaded) of cardiotoxin interacts with phospholipid tails.

**Figure 4:**
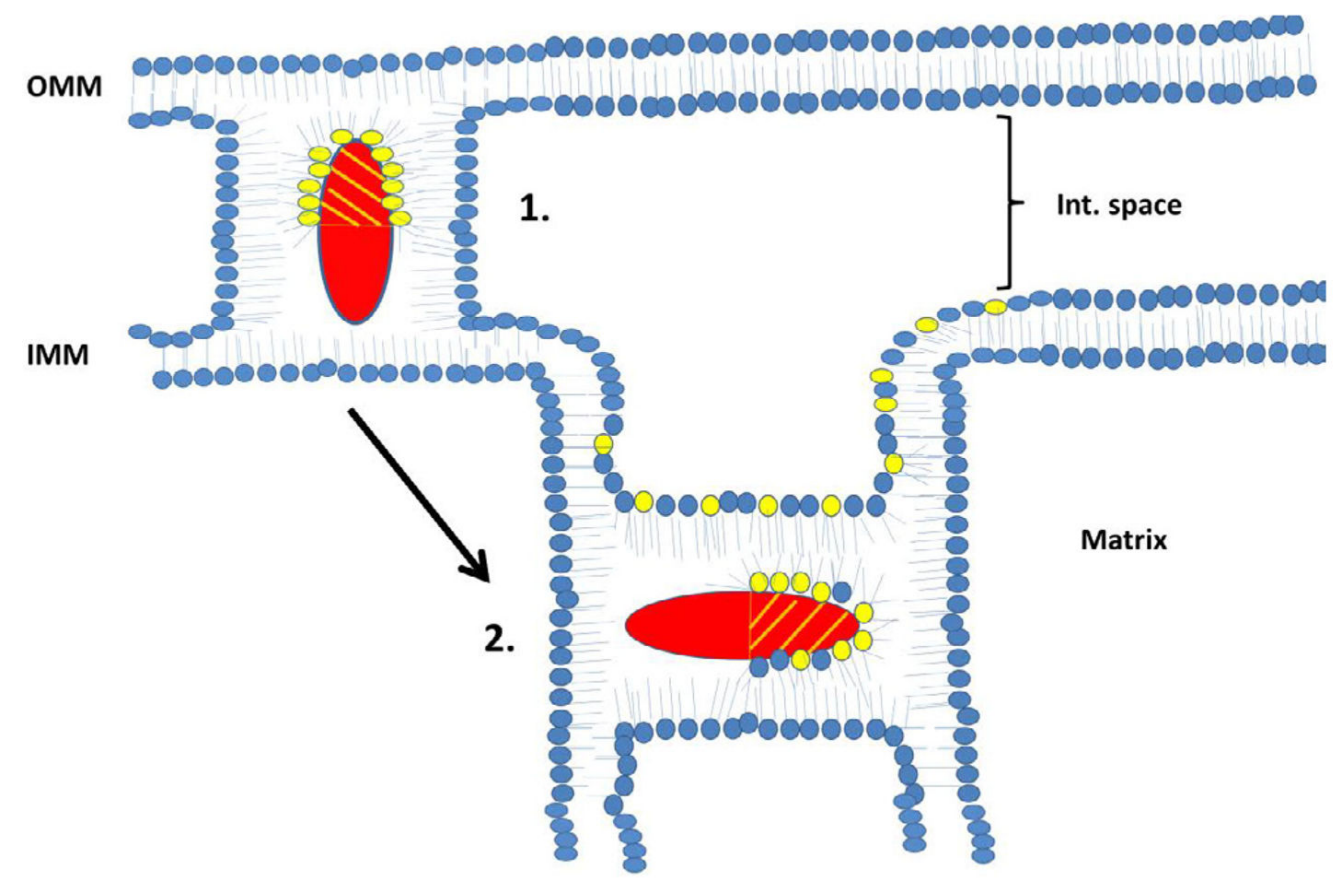
Schematic diagram on the proposed mechanism by which cardiotoxins can interact and disrupt either the OMM or the IMM of mitochondria Following its internalization into the mitochondrial intermembrane space, cardiotoxin initially sequesters CL molecules that reside at the inner leaflet of the OMM and disrupt the organization of the lipid bilayer by promoting OMM rupture and/or swelling. Cardiotoxin can also form a transient inverted micelle by interacting with the inner and outer leaflets of the OMM and IMM respectively. Formation of inverted micelles triggers the fusion of OMM and IMM membranes and the subsequent externalization of CL to the outer leaflet of OMM (not shown in this figure). In addition, cardiotoxin can translocate to the IMM and disrupt mitochondrial function by altering both cristae and IMM structure (arrow showing the progression towards step 2).

**Figure 5:**
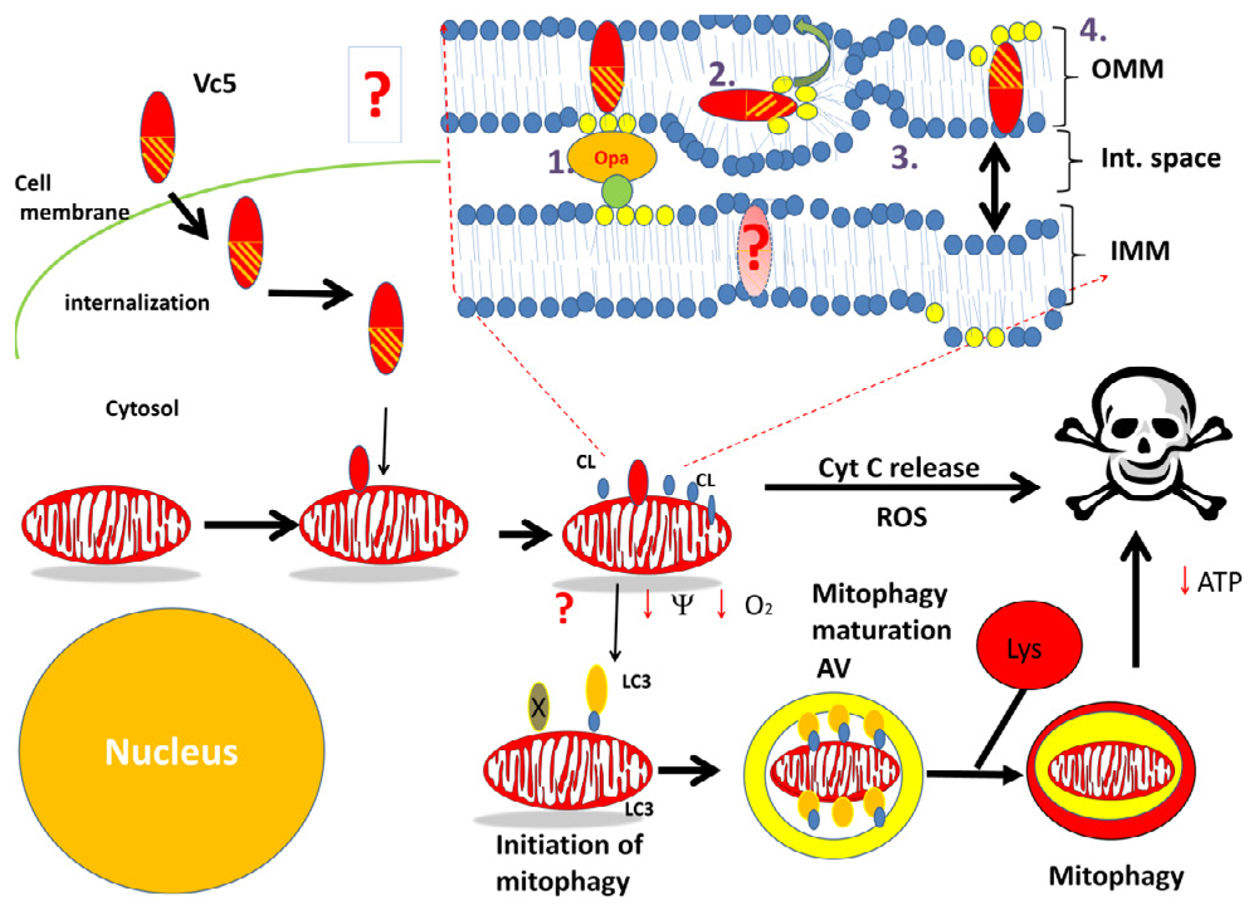
Proposed figure model describing the interaction of cytotoxins with cell membranes, and its downstream effects on mitochondrial function and survival following its internalization. Cytotoxin on the extracellular space interacts with the cell membrane of the target cell. Cytotoxin is then internalized into the cytosol through uncharacterized mechanisms and can translocate to the mitochondrion. Upon binding to the OMM, cytotoxin induces a disorganization of the phospholipid bilayer which leads to the externalization of CL, rupture of the OMM by destroying the CL contact sites that keep the IMM in contact with the OMM and required for mitochondrial integrity (formed by the interaction of IMM-localized Opa1 and other proteins with CL), and can form transient inverted micelles that can be translocated to the IMM. At the IMM, cardiotoxin can also promote a disorganization of the lipid bilayer and disrupt oxidative phosphorylation as evidenced by a loss of ATP levels and a collapse of the transmembrane potential, and fission of adjacent IMM membranes. Fragmented mitochondria can either be sequestered by autophagosomes leading to their lysosomal-mediated hydrolysis or may release cytochrome C which initiates downstream apoptotic signaling by activating the apoptosome complex. The externalization of CL to the OMM triggers the removal of mitochondria via mitophagy by facilitating the association of the autophagy receptor microtubule-associated protein 1 light chain 3 (LC3) with externalized CL. The link between cardiotoxin-mediated cytotoxicity with mitophagy has not been established yet (shown as a question mark in this model). Hence, future studies are required to determine whether treating cells with cardiotoxins can promote mitophagy in cancer cells by disrupting the OMM structure and causing the externalization of CL.

In addition to binding to PS, some cytotoxins have the ability to interact with cardiolipin (CL) *in vitro*. For instance, *N. oxiana* cytotoxins interact with liposomes enriched with CL to form non-bilayer structures which results in the externalization of CL to the outer leaflet of liposomal membranes [14,46–48]. By using EPR, ^1^H-, ^2^H- and ^31^P-NMR spectroscopic techniques, we showed that *Naja* cytotoxins form intermembrane toxin-lipid complexes that resemble inverted micelles that are characterized by an isotropic orientation of the alkyl tails of phospholipids [16,47,48]. Such atypical lipid-based complexes with the cytotoxin positioned in the center of the micelle are produced when cell membranes of two cells interact prior to membrane fusion [16] (Figure 2). These unique lipid-protein complexes (also reported in a recent study by Forouhar et al. [49]) mediate not only cell membrane fusion, but also stimulate the transient formation of pores [49]. Following membrane fusion, an inverted micelle containing cytotoxins can insert into a bilayer of the fused cell (Figure 3) with the ensuing internalization of cytotoxin into the cytosol [47] and the translocation of cytotoxin to mitochondria [30,86,87] (Figure 5). Once bound to mitochondria, a cytotoxin internalizes into the mitochondrial intermembrane space and sequesters anionic phospholipids (CL and PS) located on the inner leaflet of the outer mitochondrial membrane (OMM) and promotes the formation of transient inverted micelles (Figure 4), which trigger the fusion of the OMM with the inner mitochondrial membrane (IMM) and the externalization of CL to the outer leaflet of the OMM. Furthermore, cytotoxin induces the permeabilization of the OMM and causes the subsequent release of cytochrome C [47]. In addition, cytotoxin promotes the formation of inverted micelles between adjacent membranes of the cristae (Figure 4) (our unpublished observations). This pathological event triggers the formation of transient pores [49] which should destabilize oxidative phosphorylation. Hence, given that externalization of CL of damaged mitochondria signals for mitochondrial autophagy (mitophagy) [88], it is possible to synthesize anti-cancer recombinant versions of cytotoxin that can selectively target mitochondria, overactivate mitophagy by inducing the externalization of CL leading to a decrease in oxidative phosphorylation, and induce a loss of ATP with detrimental consequences to cancer cells (Figure 5).

## Phospholipase A_2_

Snake venoms from *Colubridae* (*sensulato*), *Elapidae*, and *Viperidae* families are rich sources of phospholipase A_2_s (PLA_2_s, phosphatidylcholine 2-acylhydrolases). These enzymes predominantly hydrolyze phospholipids containing unsaturated fatty acid tails at the *sn*-position which leads to the generation of lysophospholipids and unsaturated fatty acids [88]. These products of hydrolysis change the physical properties of cell membranes and activate downstream signal transduction pathways, which can produce widespread cellular pathology [89]. Structural studies on snake venom PLA_2_s have identified highly conserved regions in these enzymes including a conserved structural scaffold [17]. These enzymes display an impressive array of pharmacological and toxicological activities which likely originated through a process of ‘accelerated evolution’ that incorporated multiple amino acid substitutions of solvent exposed charged residues in these molecules [90]. Interestingly, there seems to be no clear relationship between the amount of enzymatic activity (lipid hydrolysis) and pharmacological action of PLA_2_ s *in vivo* [17]. Hence, this phenomenon has baffled toxinologists for decades from the view point that there is no clear correlation on structure to function or enzyme function to pharmacological action of snake venom PLA_2_s. Clinically, presynaptic-acting neurotoxic phospholipases are components of snake venom that contribute to most deaths due to snake envenomation [23]. The presynaptic neurotoxic activities of phospholipases of *Crotalid* venom have been correlated to the presence of the acidic subunit of the dimeric form of the Mojave toxin [26,91]. Given that the presence of the acidic subunit varies within and across rattlesnake species, it is imperative that anti-sera with immunodepleting activity be specifically produced against the acidic subunit of neurotoxic phospholipases [45].

There have been various attempts by different research groups to elucidate the complex pathological mechanisms induced by snake venom PLA_2_s, particularly regarding their ability to block neuromuscular transmission and induce acute muscle damage, two activities responsible for key pathological events in humans following snake bite envenoming [92]. The enzymatic and pharmacological activities of venom PLA_2_s were characterized by Rosenberg and colleagues in 1981 [93]. Their experimental approach included chemically modifying PLA_2_s of variable toxicity followed by performing a variety of biochemical assessments of the modified enzymes. In addition, this research team used a variety of *in vivo* pharmacological assays that included determining the LD_50_ *in vivo*, analyzed the effects of PLA_2_ on neuromuscular preparations, measured cardiotoxicity, coagulation, and hemolysis [93]. Overall, their findings along with other reports [94] proposed a model suggesting that PLA_2_s interact with other protein targets located in phospholipid bilayers as an alternate mechanism of toxicity that does not involve phospholipid hydrolysis. Hence, the non-catalytic mediated toxicity induced by some snake venom PLA_2_s may be regulated by other molecular regions that are distinct from the catalytic site. However, once bound to relevant targets, it is believed that enzymatic hydrolysis may play a role in membrane damage and toxicity induced by some PLA_2_s. This hypothesis was also raised by Kini and Evans [95] in 1989 who proposed that toxic PLA_2_s interact with protein targets to induce cellular pathology. This concept was initially tested by using a combination of biochemical and site-directed mutagenesis approaches [94,96,97] which led to the identification of additional ‘pharmacological sites’ in PLA_2_s from various venom sources. These presumed ‘pharmacologically active sites’, which interact with membrane proteins that are distinct from the catalytic sites, differ among various PLA_2_s in that they produce cellular pathology in a non-catalytic manner [17,95].

### The catalytically inactive PLA_2_

Venoms of some Crotalid snakes are known to contain catalytically inactive PLA_2_s which harbor a Lys in position 49 *in lieu* of a highly conserved Asp residue [98]. Nevertheless, these Lys49-PLA_2_s still display toxicity (myotoxicity) *in vivo*. Hence, like cytotoxins, this group of toxins exerts plasma membrane damage in the absence of catalytic activity. Despite initial claims that residual enzymatic activity remain in these variants of snake venom PLA_2_, it was shown that Lys49-PLA_2_s are indeed devoid of catalytic activity due to their inability to bind Ca^2+^, a key cofactor that is critical for PLA_2_-mediated lipid hydrolysis [17]. In addition, studies performed by Lomonte et al. 1994 [94] led to the identification of several structural regions that mediate cytotoxicity of Lys49-PLA_2_s. One region is composed of hydrophobic and cationic amino residues (amino acids 115–129) that are located near the C-terminal region of the molecule [94]. Further site-directed mutagenesis and molecular biology studies [97] corroborated the relevance of the C-terminal cationic-hydrophobic segment for the membrane-disrupting activity of these toxins, which likely underlies myotoxicity of Lys49 PLA_2_s.

The proinflammatory *in vitro* effects of another Lys49-PLA_2_ homologue, BaltTX-I from *Bothrops alternatus* venom, were investigated on thioglycollate-elicited (TG) macrophages [99]. At non-cytotoxic concentrations, BaltTX-I stimulated complement receptor-mediated phagocytosis and induced superoxide production in TG-macrophages. Moreover, PKC is involved in the enhancement of complement-mediated phagocytosis induced by BaltTX-I since treating cells with staurosporine, a selective inhibitor of PKC, abolished this activity and also suppressed superoxide production. Hence, treating macrophages with BaltTX-I at non-cytotoxic concentrations can upregulate complement-mediated phagocytosis in macrophages and cause non-specific inflammation [99].

### Non-catalytic functions of PLA_2_

Lambeau and colleagues in 1989 identified membrane proteins located in neuronal cells (“N-type PLA_2_ receptor”) [100] and myogenic cells (“M-type PLA_2_ receptor”) [99] that can bind with high affinity to toxic PLA_2_s. The M-type muscle receptor was further characterized and was shown to specifically bind to the PLA_2_ toxins with dissociation constants within the picomolar range [101]. This receptor (~180 kDa) was purified to homogeneity by using affinity chromatography and was found to bind with high affinity to PLA_2_ in liposomes [101]. In addition, by performing molecular biology techniques [102], the authors of this study demonstrated that certain snake venom PLA_2_s can unambiguously bind to proteins, in addition to binding to phospholipid membranes [17]. Overall, these experiments raised the possibility that PLA_2_s can exert multiple pathological effects in cells by not only actin as lipid-hydrolyzing enzymes, but by also serving as ligands for specific surface-exposed membrane proteins.

Numerous studies have been devoted to characterizing the myotoxic effects of various PLA_2_s in skeletal muscle [17]. For instance, myotoxic viperid PLA_2_s generally induce local tissue damage which, in conjunction with the action of hemorrhagic metalloproteinases, generate complex and widespread tissue pathology. Myotoxic elapid PLA_2_s and some viperid PLA_2_s provoke systemic myotoxicity that is associated with myoglobinuria and acute renal failure [17]. The molecular mechanisms of toxicity induced by myotoxic venom PLA_2_s include their ability to disrupt the sarcolemmal membranes which results in a loss of ion gradients and induces massive Ca^2+^ influx associated with the onset of several irreversible degenerative processes [103,104], a phenomenon that was characterized by Dixon et al, 1996 [105]. In brief, by employing immunohistochemistry and immunoelectron microscopy techniques, the authors of this study showed that notexin, a myotoxic PLA_2_ from *Notechis scutatus*, binds specifically to the sarcolemma suggesting that specific unknown binding proteins exist in the plasma membrane of skeletal muscle fibers [105]. Upon binding to the sarcolemma, notexin induces the enzymatic hydrolysis of sarcolemmal phospholipids which results in membrane damage and Ca^2+^ influx, followed by hypercontraction of myofilaments, and induction of other downstream degenerative events [105]. Owing to the similar pathological features described for other myotoxic PLA_2_s derived from viperid and elapid venoms, it is likely that a similar non-catalytic mechanism of action operates in all of PLA_2_s [17]. Hence, future research on catalytically inactive PLA_2_s is critical to further advance our understanding of the pathological contribution of this poorly characterized class of snake venom PLA_2_s.

### Neurotoxic snake venom PLA_2_s

**Figure 6:**
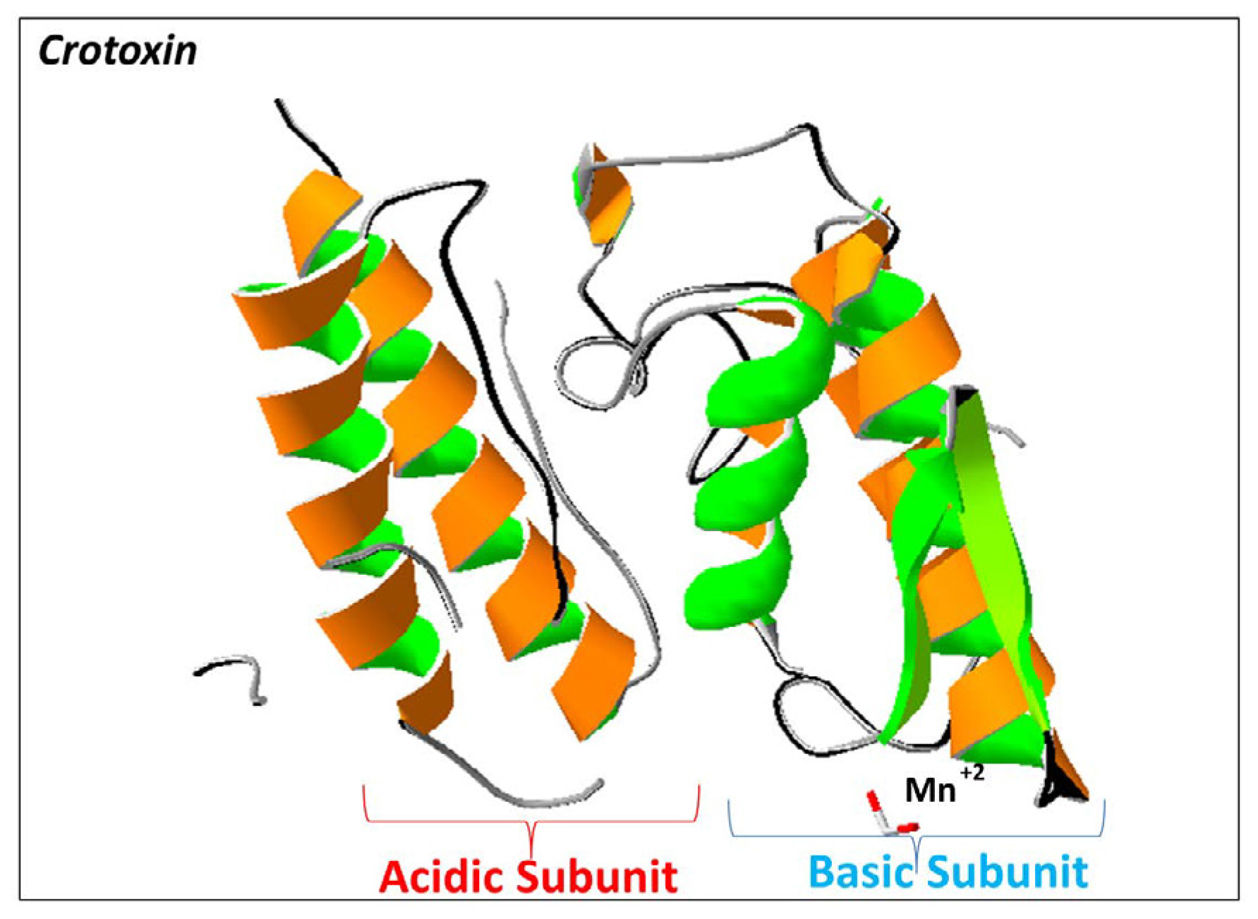
A rendering of the 3D structure of crotoxin from *Crotalus durissus terrificus* A ribbon diagram of the dimeric crystal structure of crotoxin (PDB #3R0L) is illustrated to highlight the acidic subunit (left side) and to show the basic subunit (right side). 3D molecular rendering and annotations of crotoxin were performed by using the Swiss PDB Viewer software.

The precise molecular mechanisms by which presynaptically-acting venom PLA_2_s (β-neurotoxins) cause neurotoxicity in snakebite victims has been the subject of high interest for several decades. Presynaptically-acting venom PLA_2_s can cause degeneration of the motor nerve terminals [106]. Structurally, neurotoxic PLA_2_s such as crotoxin from *Crotalus durissus terrificus* consists of an acidic subunit which targets the enzyme complex to the synaptic cleft, and a basic subunit which catalyzes the hydrolysis of phospholipids upon binding to specific synaptic proteins (Figure 6). However, the question of how these PLA_2_s are targeted to the synaptic cleft remains to be elucidated. A recent study partially addressed this question by characterizing the presynaptic effects of an ultrastructurally traceable modified version of ammodytoxin A (a β-neurotoxin), a neurotoxic protein of *Vipera ammodytes* [107]. In brief, this toxin-nanogold conjugate was intramuscularly injected in mice and their soleus muscles were isolated at different time points for ultrastructural analyses. The electron micrographs showed that this β-neurotoxin was internalized into the motor nerve terminal, followed by its translocation to mitochondria and into vesicular structures [107]. This work suggests that the β-neurotoxins are selectively targeted to synapses of motor neurons, where they are internalized at the synaptic boutons to block neurotransmission at the neuromuscular junction through poorly characterized molecular mechanisms. Other studies that utilized mass spectrometry techniques suggest an alternate toxic mechanism for β-neurotoxins. This model involves the hydrolysis of phospholipids at the plasma membrane after its association with specific protein target(s) in neurons [8]. Additionally, β-neurotoxins can induce the swelling of the axons and dendrites in neurons and this pathological event was associated with a robust Ca^2+^ influx and mitochondrial pathology induced by phospholipid hydrolysis of mitochondrial membranes [9]. Furthermore, β-neurotoxins bind specifically to mitochondria and induce the opening of the permeability transition pores in mitochondria which stimulate downstream apoptosis [9].

An unusual PLA_2_, termed bitanarin, was recently isolated from puff adder from *Bitis arietans* venom. This enzyme blocked the response of *Lymnaea stagnalis* neurons to acetylcholine (ACh) and competed with ^125^I-α-bungarotoxin for binding to neuronal α7 and muscle-type nicotinic ACh receptors (nAChRs) as well as to Ach binding protein from *L. stagnalis* [108]. PLA_2_s from various snake venoms can interact directly with nAChRs. For instance, PLA_2_s from *Vipera ursinii*, *Naja kaouthia*, and *Bungarus fasciatus* suppressed acetylcholine-mediated currents in neurons from *L. stagnalis* [109]. This effect was evident at PLA_2_ concentrations in the micromolar range. Hence, it is likely that these PLA_2_ molecules have a domain that interacts with nAChR and that the pharmacological suppression of nAChRs may underlie the non-catalytic toxic actions of these types of PLA_2_s [109].

### Molecular mechanisms of PLA_2_-mediated catalysis

In contrast to many water soluble enzymes, PLA_2_s are unique in that they preferentially catalyze aggregated substrates including phospholipid monolayers, liposomal bilayers, and micelles through a phenomenon known as ‘interfacial activation’[17,19]. To understand the molecular mechanism of interfacial activation, Sigler and colleagues [19,110] studied the 3D structure of PLA_2_ from *Naja atra* cobra venom complexed with a phosphonate transition state analogue and compared it to the 3D structure of the uncomplexed version of cobra PLA_2_. Overall, they found that the interfacial binding surface is located in the vicinity of the mouth of the hydrophobic channel. The interaction of PLA_2_ with its substrate occurs inside a hydrophobic internal surface that is shielded from the bulk solvent. Following the transfer of substrate to the active site in the absence of solvation, PLA_2_-mediated catalysis involves the removal of a proton from a water molecule by a His48 located at the catalytic site which is further stabilized by Asp99 through the formation of a tetrahedral intermediate. Furthermore, the geometry of the tetrahedral intermediate is coordinated by Ca^2+^. This step is followed by a productive collapse of the tetrahedral intermediate to yield hydrolysis products. Furthermore, the *sn*-1 and *sn*-2 alkyl groups of the phospholipid substrate are positioned in parallel to the hydrophobic channel that extends from the catalytic site to the surface of the molecule close to the N-terminus. Hence, it is likely that catalysis cannot occur in the case of dispersed phospholipids but only in an aggregated interface of phospholipids.

Pathologically, recent work by Montecucco et al. 2005 have demonstrated that the PLA_2_ hydrolysis products, including oleic acid and lysosphosphatidylcholine, are sufficient for inducing neurotoxicity. Indeed, injecting mice with different mixtures of lysophospholipids (PLA_2_ hydrolysis products) phenocopy the effects of injecting mice with a mixture of presynaptic snake venom neurotoxins [111]. These results suggest that changes in the membrane curvature, lipid asymmetry, and local changes of phospholipids at the synapse induced by snake venom PLA_2_s contribute to neurotoxicity. Hence, the non-toxic targeting subunit of neurotoxic dimeric PLA_2_s likely synergizes these neurotoxic effects by localizing the membrane-altering activities of PLA_2_s at the synapse.

Our previous work on the lipid binding properties of PLA_2_s derived from *Crotalus m. molossus* and *Naja oxiana* venoms [14,15,18,37] has shown that the lipid hydrolytic activities of PLA_2_s are sensitive to minor structural differences and to phospholipid content of the lipid interfaces located on the cell membrane. For instance, lipid bilayers composed of only phosphatidylcholine (PC) were highly susceptible to lipid hydrolysis by neutral and acidic PLA_2_s, whereas lipid bilayers containing a high amount of PC and a low amount of acidic phospholipids were slightly susceptible to PLA_2_s-mediated lipid hydrolysis. The molecular organization of phospholipid interfaces can be significantly altered by basic membrane-active peptides (MAP). Cell membranes enriched in PC and that were treated with MAPs were found to contain a tightly packed lipid interface which inhibited the catalytic activity of PLA_2_s. On the other hand, phospholipid membranes containing acidic phospholipids and PC that were treated by MAP showed various interfaces (inverted micelles and various types of non-bilayer structure) and increased the catalytic activities of PLA_2_s. Therefore, it is likely that phospholipase activity is also controlled by endogenous MAPs. Indeed, several endogenous MAPs such as lipocortin-like protein [112] and cationic peptides isolated from human arthritic synovial fluid [113] modulate the catalytic activities of endogenous phospholipases. Hence, a further understanding on the molecular mechanism(s) by which PLA_2_s promote hydrolysis of cell membranes has very important underlying biomedical implications for the treatment of autoimmune disorders. Overall, elucidating the transient structural changes on the molecular organization of phospholipid interfaces induced by PLA_2_s in cell membranes should aid the design of basic peptides that suppress overactive endogenous PLA_2_s as seen in various autoimmune disorders and in chronic inflammatory conditions including rheumatoid arthritis, asthma, and possibly in some neurodegenerative diseases [18].

## Zn^2+^-dependent metalloproteinases

Snake venom metalloproteinases (SVMPs) are major components of most *Crotalid* and *Viperid* venoms [21]. These enzymes - the key contributors to lethal toxicity in these venoms - are large multi-domain proteins that are classified as P-I, P-II, and P-III based on the presence or absence of non-catalytic ancillary domains that extend beyond the mature proteinase domain. While P-I (~20–30 kDa proteins) have only a proteinase domain, the P-II SVMPs (~30–60 kDa) contain proteinase and disintegrin domains [114,115]. Furthermore, the P-III SVMPs (~60–100 kDa) contain a proteinase, a disintegrin, and cysteine-rich domains. For efficient catalysis, the catalytic domains of SVMPs require association with divalent cations such as Zn^2+^ at a 1:1 M ratio and the N-terminal and C-terminal domains bind to Ca^2+^ [116]. While Zn^2+^ is required for catalytic activity of SVMPs, Ca^2+^ is involved in the structural stabilization of the SVMPs and allows for modest localized structural contractions of the molecule [117]. The active site of the P-I domain contains a well conserved “HEXXHXXGXXHD” amino acid sequence that harbors the obligatory catalytic His triad and a portion of the Met-turn. The “Met-turn” structure contains a conserved Met residue that forms the hydrophobic basement (lower lip) of the catalytic site [21].

The SVMPs produce variable tissue and cellular pathology in bite victims. The majority of SVMPs induce profuse hemorrhage, blood coagulation, and the inactivation/activation of complement proteins [118–120]. Other members of the SVMPs possess fibrin(-ogen) olytic activity [120,121], act as prothrombin activators [122,123], contain pro-apoptotic activity [124], serve as in activators of blood serine proteinase inhibitors [125,126], and activate factor X (a pro-coagulation protein) [127,128]. The high variation in physiological activities exhibited by SVMPs is likely due to modest structural differences of these enzymes located at interconnecting loops that precede or follow the Met turn and of their northern cleft walls which form the roof of the catalytic cleft [21,115]. Moreover, the ancillary domains of SVMPs may not only significantly increase hemorrhagic potency of these enzymes, but may also direct the P-I domain to various target sites [129]. Clinically, there are two potent anti-sera that have been developed and tested in clinical trials for treating snakebite victims due to envenomation by *Echis* and *Naja* snakes, a serious health concern in sub-Saharan Africa [130,131]. These two anti-venoms can stop the profuse hemorrhage induced by SVMPs of certain *Naja* and *Echis* species [130–132]. Mechanistically, SVMPs in *Naja* venoms can be efficiently neutralized by immunodepletion by certain anti-sera used for treating snakebite victims [130].

### Hemorrhagic SVMPs

The robust proteolytic degradation of capillary basement membrane proteins and the leakage of blood components from the vasculature into surrounding tissues underlie the hemorrhagic activity of the SVMPs [21,133]. Most SVMPs can produce systemic hemorrhage *in vivo*. Indeed, immediately following the intramuscular injection of purified snake venom fractions containing SVMPs in mice, extensive hemorrhage within the affected muscle tissue and adipose cells has been documented [134,135]. The pathophysiological spectrum induced by SVMPs also includes the formation of platelet plugs in blood vessels, the swelling and disruption of endothelial cells in capillaries, whereas other blood vessels shrivel and get disrupted. Interestingly, blood vessels containing intact intercellular junctions can still contain damaged endothelial cells and either a disorganized or lack of the basal lamina [134,135].

Laminin, nidogen, and type IV collagen in muscle tissue are proteolytically degraded by hemorrhagic SVMPs as immunofluorescence quantitation of these basement membrane proteins reveal a significant reduction in the levels of all of these proteins [133]. Hemorrhage is most likely the combined result of the proteolytic action of the SVMPs on basement membrane proteins and mechanical damage induced by hemodynamic forces [133]. Furthermore, snake venom PLA_2_s may exacerbate hemorrhage by destabilizing the basement membranes following the hydrolysis of phospholipids [136].

### Fibrinolytic SVMPs

A variety of pit viper venoms contain fibrin(ogen) olytic activity [137]. However, the level of this proteolytic activity is highly variable even within a particular genus of snakes [138]. One of the most potent fibrinolytic enzymes is fibrolase, a P-I metalloproteinase isolated from southern copperhead *Agkistrodon contortrix* venom [139,140]. Fibrolase cleaves both the Aα- and the Bβ-chains of fibrinogen, while having no effect on the γ-chain. Fibrolase acts directly on fibrin and does not rely on the activation of plasminogen as fibrolase neither activates nor degrades plasminogen [139]. Remarkably, the blood serine protease inhibitors have no effect on fibrolase [141], but it is inhibited by human α2 macroglobulin (α2-M) through molecular trap mechanism that involves the formation of an irreversible covalent bond with the proteinase [142,143]. Interestingly, the larger P-III SVMPs do not fit into this molecular trap and are therefore not inhibited by α2-M [143].

Fibrolase effectively dissolves carotid arterial thrombi in canines and in other animals [144]. The clinical potential of a recombinant truncated form of this enzyme (composed of residues 3–203 of wild-type fibrolase) was tested in clinical trials for treating abnormal blood clot formation [144]. This recombinant protein, termed alfimeprase, behaved identically as wild-type fibrolase in terms of enzymatic activity and inhibition by α2-M [144]. *In vivo* studies with alfimeprase have shown that the enzymeis able to degrade blood clots up to six times faster compared to recombinant tissue plasminogen activators [144]. A significant advantage of using alfimeprase over plasminogen activators lies in the fact that α2-M inhibits the proteolytic activity of alfimeprase, thereby preventing the potential for unwanted profuse systemic bleeding due to dysregulated thrombolysis induced by alfimeprase. Alfimeprase has been shown to effectively dissolve the thrombic clots when delivered to the site of the thrombus in acute limb ischemia and is readily inactivated by α2-M [144,145]. Unexpectedly, although alfimeprase passed Phase 1 and 2 clinical trials, it failed Phase 3 trials as treating patients with the drug was unable to meet several biological end-points including peripheral arterial occlusion [39,146,147].

### Apoptosis-inducing SVMPs

When studying the apoptotic activity of vascular apoptosis-inducing protein 1 (VAP1) from *C. atrox* venom in vascular endothelial cells, Araki et al. 2002 [124] reported that VAP1, a homodimeric protein of the P-III SVMPs, promotes apoptosis by interacting with integrins rather than by blocking the adhesion of vascular endothelial cells or by directly degrading the extracellular matrix proteins. Although VAP1 and monomeric VAP2 [148] are weakly hemorrhagic toxins [149], the findings shown by Araki et al. 2002 [124] suggest that the disintegrin domain of the P-III SVMP must be at least partly involved for promoting apoptosis. Furthermore, the authors of this study suggested that α3, α6 and β1 integrins are involved in mediating VAP1-induced apoptosis as antibodies specific to these integrins inhibited cell death. Although an interesting area of research, further studies need to be conducted in order to fully define the molecular mechanism(s) that regulate VAP1/2-mediated apoptosis.

### Prothrombin-activating SVMPs

There have been two groups of prothrombin-activating SVMPs that have been identified in snake venom: Group A and Group B, which are structurally unrelated to the human prothrombin activators - the blood coagulation serine proteinases [122]. The group A activators require no cofactors, such as Ca^2+^, phospholipids or other proteins, for activation of prothrombin. The Group A prothrombin-activating SVMPs are predominantly found in viper venoms [150]. The best characterized Group A activator is ecarin isolated from *Echis carinatus* venom [150]. This enzyme is a P-III SVMP that contains metalloproteinase, disintegrin, and cysteine-rich domains. Ecarin consists of 426 residues that shows a high sequence homology to Russell’s viper venom FX activator heavy chain (64% identity) [151]. The P-I domain of ecarin has a consensus Zn^2+^-binding active site HEXXHXXGXXHD and an RDD sequence *in lieu* of the canonical RGD sequence located within the disintegrin domain [124,151]. The pro-thrombin activating SVMPs produce coagulation by proteolytically cleaving the Arg320-Ile321 bond in thrombin which actives an enzyme called meizothrombin [152], which appears to consist of a B-chain and an extended A-chain [124,150]. Meizothrombin is inherently unstable and eventually is auto-catalytically converted to thrombin.

Group A prothrombin activators have been isolated from the venom of *Bothrops* species and exhibit very similar biochemical properties to ecarin, including the production of meizothrombin [153]. Another Group A prothrombin activator from *B. erythyromelas* venom, named berythractivase, did not elicit a hemorrhagic response even though its primary structure is related to that of snake venom hemorrhagic metalloproteinases and are functionally similar to the Group A prothrombin activators [153]. This lack of hemorrhagic response by this prothrombin activator distinguishes it from most of the Group A prothrombin activators.

Group B prothrombin activators appear to form a heterodimeric complexes [21]. One of them is carinactivase-1 from *E. carinatus* venom. Carinactivase-1 consists of two subunits held tightly through non-covalent bonds. One subunit (62 kDa) has metalloproteinase activity and is homologous to the single-chain enzyme ecarin, while the other subunit (31 kDa), which appears to act as regulatory subunit, is a C-type lectin-like disulfide-linked dimer composed of polypeptides of 17 and 14 kDa [154]. The activation of prothrombin by carinactivase-1 requires Ca^2+^ at millimolar concentrations. Carinactivase-1, unlike ecarin, does not activate prothrombin derivatives in the absence of Ca^2+^. The following mechanism for the activation of prothrombin by carinactivase-1 has been proposed in the following manner: once the regulatory subunit of carinactivase-1 binds to the Ca^2+^ bound Gla domain in prothrombin, the 62 kDa catalytic subunit cleaves the bond between the A and B chains of prothrombin. Thus, the 31-kDa regulatory subunit of carinactivase-1 acts as an effective targeting domain for the substrate and reduces the apparent K_m_ for the SVMP [154,155].

### Factor X-activating SVMPs

Physiologically, the proteolytic activation of blood coagulation Factor X (FX) to FXa occurs via cleavage of the Arg52-Ile53 bond found in the heavy chain of human FX [128]. Remarkably, FX can be activated by SVMPs. FX-activating SVMPs are found in venom from *Viperidae* and *Crotalidae* [21]. One well characterized FX activator (VLFXA) was isolated from the venom of *Vipera lebetina* [127,128]. This FX activator is a P-III SVMP composed of metalloproteinase, disintegrin, and cysteine-rich domains. Structurally, VLFXA is a glycoprotein composed of three chains, a heavy chain of 57.5 kDa and two light chains of 17.4 kDa and 14.5 kDa respectively, and all these chains are stabilized by disulfide bonds. The light chains contain 123 and 135 residues and both belong to the class of C-type lectin-like proteins [128]. VLFXA has no effect on fibrinogen, prothrombin, and plasminogen, suggesting that VLFXA activates FX specifically, although it activates factor IX (FIX) as well [127]. Mechanistically, VLFXA cleaves the Arg52-Ile53 bond in the heavy chain of human FX and the Arg226-Val227 bond in human FIX [127]. Hence, the FX-activating SVMPs primarily recognize the Ca^2+^-bound conformation of the Gla domain in FX via the regulatory subunit (presumably a cysteine-rich domain), and the catalytic subunit subsequently converts FX to active FXa [127,156]. Unlike fibrolase, there have been no attempts to this date to develop VLFXA for the treatment of hemophilia or other blood clotting deficient disorders.

### Structure of SVMPs

To date, the high resolution X-ray structures of nine P-I and seven P-III SVMPs have been solved which have shed valuable insight into the general topology and 3D structure of SVMPs [129]. The 3D structures of SVMPs show that the proteinase domain has a similar topology to that of mammalian A Disintegrin and Metalloproteinases (ADAMs) [129,157]. Beyond the proteinase domain, the disintegrin domain (D) in PIII metalloproteinases is divided into D shoulder (Ds) and D arm (Da) components. The Ds extend from the metalloproteinase domain (M) in an opposite orientation relative to the catalytic site. The D domain together with the cysteine-rich (C) domain form a concave surface that faces the M-domain. Unlike the RGD sequence contained within “genuine” snake venom disintegrins, the disintegrin-loop in some P-III SVMPs appears to be inaccessible for protein binding [21]. Although it was proposed that structural differences in the D and C domains could be involved in mediating substrate specificity [158], the functional roles of the D and C domains of the P-III SVMPs have not been clearly elucidated [159].

Adamalysin II from *Crotalus adamanteus* venom was the first P-I SVMP whose crystal structure was solved at high resolution (~2.0 Ǻ) and is commonly used as a template structure to model the topology of SVMPs whose crystal structures have not been solved [160,161]. Overall, adamalysin II is an ellipsoidal molecule with a flat active-site cleft that separates the “upper” part of the molecule from the “lower” domain which consists of 50 amino acid residues that are folded into variable loop regions, a conserved C-terminal α-helix, and an extended C-terminal tail that associates with the ‘upper” domain via a disulphide bond. The “upper” domain is composed of a highly twisted five-stranded β-sheet flanked by long and short α-helices, one of which acts as the central “active-site helix”. The catalytic Zn^2+^ is located at the bottom of the active-site cleft and is coordinated by His^142^, His^146^ and His^152^, and by H_2_O bound to Glu^143^. The long active site helix ends with Gly^149^ where it turns sharply towards His^152^. The conserved Met^166^ of the “Met-turn” forms a hydrophobic surface that acts as the base for the three Zn^2+^-binding imidazoles [161].

SVMPs can produce variable hemorrhagic activity *in vivo*. A recent study proposed a molecular mechanism that gives rise to differences in hemorrhagic activities of SVMPs [162]. By employing sophisticated molecular dynamic simulations, the authors of this study found significant differences in the flexibility of the interconnecting loops that precede and that follow the Met turn. In brief, the hemorrhagic SVMPs show high flexibility of the loop that is composed of residues 156–165 while the non-hemorrhagic SVMPs have a very dynamic loop which follows the Met turn (amino acid residues 167–175). Hence, it appears that the flexibility of these two surface-exposed loops seems to be a requirement for the hemorrhagic activity of SVMPs. Moreover, the higher flexibility of these two loops may confer differences in substrate specificity for P-I SVMPs [162]. Furthermore, by comparing the crystal structure of Bap1 (a P-I SVMP from *Bothrops asper*) with other hemorrhagic and non-hemorrhagic PI SVMPs [163], the authors of this study showed that an interconnecting loop, formed by amino residues 153–176, show significant structural deviations among SVMPs. This interconnecting loop lies close to the catalytic active-site and may influence the interaction of the SVMPs with extracellular proteins of the basement membrane of capillaries [163]. Another study that employed several bioinformatics and proteomics tools successfully classified up to 19 hemorrhagic and non-hemorrhagic PI metalloproteinases on the basis of their total polar molecular surface area. In brief, the authors of this study demonstrated that hemorrhagic metalloproteinases tended to contain a significantly higher total polar molecular surface area compared to non-hemorrhagic metalloproteinases suggesting that surface polarity plays a very crucial role for conferring hemorrhagic activities to PI-type SVMPs.

The intra-species variation in biochemical activities of SVMPs is a highly intriguing phenomenon that has puzzled toxinologists for several decades. Recently, we have shown a genetic basis for the intra-species variation in metalloproteinase-associated activities observed for rattlesnake venom of the *Crotalinae* genus [25]. In brief, we found that the proteolytic and hemorrhagic differences observed in rattlesnake venom from various *Crotalus scutulatus scutulatus* (Mojave) rattle snakes was correlated with the presence of four different metalloproteinase genes termed GPI, GPII, GPIII, and GPIV. In summary, the venom from Mojave rattlesnakes containing the GPI metalloproteinase gene possessed high hemorrhagic and high proteolytic activity while the venom of rattlesnakes containing the GPII gene lacked both hemorrhagic and proteolytic activities but was able to degrade fibrinogen [25]. More recently, our structural analyses of crystal structures and of molecular models deduced from the amino acid sequences of the four groups of metalloproteinase genes (GPI-GPIV) of *Crotalus scutulatus scutulatus* and from *Crotalus atrox* (atrolysins A-E) suggest that the intra-species variation in hemorrhagic activities of metalloproteinases may be a consequence of a combination of structural and physico-chemical factors including differences in backbone flexibility, spatial conformational differences in the northern cleft wall, and the presence of specific negatively charged residues that surround the catalytic groove [164]. Finally, molecular dynamics and field potential maps for each *C. s. scutulatus* metalloproteinase model demonstrated that the non-hemorrhagic metalloproteinases (GP2 and GP3) contain both a highly positively charge field potential surfaces while the hemorrhagic metalloproteinases GP1 and atrolysin C showed extensive acidic field potential maps [164].

Snake venom contains a panoply of metalloproteinases with diverse biochemical activities. To this end, it is possible to identify biomedically relevant enzymes for treating neurodegenerative diseases. Certain oligomeric forms of highly phosphorylated α-synuclein are highly resistant to chymotrypsin or to proteinase K treatment [165,166]. As Parkinson’s disease patients are characterized by the accumulation of phosphorylated extracellular protein aggregates termed Lewy bodies [167] or senile plaques in Alzheimer’s disease patients, it is conceivable that these highly phosphorylated protein aggregates may be proteolytically cleaved by basic non-hemorrhagic metalloproteinases to help slow the progression of the disease. Hence, by applying sophisticated molecular modeling techniques, affinity docking simulations in combination with biochemical, and *in vivo* testing, it is possible to identify basic non-hemorrhagic (i.e., Adamalysin II) but highly proteolytic SVMPs that can be further developed as novel anti-neurodegenerative agents for dissolving extracellular protein aggregates.

## Conclusion

Cytotoxins, phospholipases A_2_s, and Zn^2+^-dependent SVMPs represent major classes of proteins found in snake venoms. Many of these proteins are responsible for producing severe pathophysiological events following envenomation and therefore represent a significant hazard for snakebite victims. Clinically, snake envenomation is very prevalent and contributes to a high mortality rate in developing countries. Moreover, it is disappointing that the lack of sufficient anti-venom supplies and clinics has not been addressed in developing countries. Hence, it is imperative that more funding and research is dedicated towards finding more efficient anti-venom and alternative treatments in order to significantly decrease the number of deaths due to snakebites.

On the other hand, many of these proteins possess pharmacologically relevant properties that can be used for treating a variety of human disorders and conditions including hemophilia, deep vein thrombosis, cancer, autoimmune disorders, and neurodegenerative diseases. For instance, cobra venom cytotoxins are widely studied for their anti-cancer properties. Moreover, research studies on the molecular mechanisms that modulate PLA_2_-associated activities may be useful for designing drugs to suppress over-activity of endogenous PLA_2_ associated with various inflammations and autoimmune disorders. Finally, SVMPs represent promising candidate drugs for treating diseases involving abnormal blood clot formation and for reversing brain pathology as observed for some neurodegenerative disorders. In summary, there is a huge untapped pharmacological potential found in rattlesnake venom that is waiting to be exploited by pharmaceutical companies that can lead to the development of new therapeutic agents for the treatment of different human diseases and disorders.
